# An efficient geometric approach to quantum-inspired classifications

**DOI:** 10.1038/s41598-022-12392-1

**Published:** 2022-05-24

**Authors:** Roberto Leporini, Davide Pastorello

**Affiliations:** 1 Department of Economics, University of Bergamo, via dei Caniana 2, 24127 Bergamo, Italy; 2 Department of Information Engineering and Computer Science, University of Trento, via Sommarive 9, 38123 Povo, Italy

**Keywords:** Computer science, Quantum information

## Abstract

Optimal measurements for the discrimination of quantum states are useful tools for classification problems. In order to exploit the potential of quantum computers, feature vectors have to be encoded into quantum states represented by density operators. However, quantum-inspired classifiers based on nearest mean and on Helstrom state discrimination are implemented on classical computers. We show a geometric approach that improves the efficiency of quantum-inspired classification in terms of space and time acting on quantum encoding and allows one to compare classifiers correctly in the presence of multiple preparations of the same quantum state as input. We also introduce the nearest mean classification based on Bures distance, Hellinger distance and Jensen–Shannon distance comparing the performance with respect to well-known classifiers applied to benchmark datasets.

## Introduction

The mathematical formulation of quantum mechanics can be used to devise machine learning algorithms that do not require any quantum hardware in the sense that the quantum formalism is applied to define data representations that are managed by classical computers. The so-called *quantum-inspired machine learning* is based on particular kinds of information storing and processing defined by means of the mathematical objects from the quantum theory that do not necessarily relates to physical quantum systems. This work is devoted to study some quantum-inspired classification algorithms from a geometric perspective and their comparison with well-known classical classifiers.

An interesting quantum-inspired binary classification algorithm has been introduced in terms of a nearest mean classifier based on the trace distance between density operators encoding feature vectors^1^. Another proposed quantum-inspired classifier is based on the Helstrom quantum state discrimination^2^ used for binary classification^3^. Both algorithms are structured on an encoding of the feature vectors into density operators and on techniques for estimating the distinguishability of quantum states like a distance in the space of the quantum states and the Helstrom measurement. Classification accuracy of these quantum-inspired classifiers can be improved by increasing, in terms of tensor products, the number of copies of the quantum states that encode the feature vectors, at the cost of dramatically increasing the computational space and time. However, in the present work, we argue that the geometric approach for representing data into quantum states provides a description of the quantum encoding that allows to implement feature maps saving space and time resources.

In this paper, we introduce the quantum encoding in terms of Bloch vectors applied to the execution of some quantum-inspired classifiers. In particular we run the Helstrom classifier representing data with different quantum encodings (i.e. different feature maps), then we define quantum-inspired nearest mean classifiers using Bures, Hellinger and Jensen–Shannon distances. In the experimental part, we present a comparison of the performances of the quantum-inspired classifiers against well-known classical algorithms.

The work is structured as follows: In “Quantum encoding” section , we introduce the representation of density operators in terms of Bloch vectors in arbitrary dimension and the basics of quantum encoding. “Quantum-inspired classifiers” section is a short description of the considered quantum-inspired algorithms that are the Helstrom classifier and the nearest mean classifiers based on several operator distances. In “Geometric approach to quantum-inspired classifications” section, we discuss how the encoding of feature vectors into Bloch vectors is useful to obtain a data representation that scales efficiently increasing the dimension of the feature space. In this section we define the classifiers based on Bures, Hellinger and Jensen–Shannon distances. In “Method and experimental results” section, there are the experimental results obtained running the quantum-inspired classifiers and the comparison with classical algorithms over some benchmark dataset. In “Conclusions” section, we draw the conclusion remarking the impact of adopting the geometric viewpoint in devising novel classification algorithms based on quantum structures.

## Quantum encoding

A *quantum encoding* is any procedure to encode classical information (e.g., a list of symbols) into quantum states. In this paper, we consider encodings of vectors in ${\mathbb {C}}^n$ and ${\mathbb {R}}^n$ into density matrices on a Hilbert space ${\mathsf {H}}$ whose dimension depends on *n*, in particular we use different quantum encodings to implement different feature maps for data representation.

The set of density matrices on the (finite-dimensional) Hilbert space ${\mathsf {H}}$ is given by ${\mathfrak {S}}({\mathsf {H}})=\{\rho \in {\mathscr {B}}^+({\mathsf {H}}): {\mathrm{tr}}\rho =1\}$, where ${\mathscr {B}}^+({\mathsf {H}})$ is the set of positive semidefinite operators on ${\mathsf {H}}$. The set ${\mathfrak {S}}({\mathsf {H}})$ is convex and its extreme elements, the pure states, are rank-1 orthogonal projectors. A pure state has general form $\rho =\left \vert {\psi } \right \rangle \left \langle {\psi }\right \vert$, and it can then be directly identified with the unit vector $\left \vert {\psi } \right \rangle \in {\mathsf {H}}$ up to a phase factor. The bases of the real space of Hermitian matrices on ${\mathbb {C}}^d$ can be used to decompose density matrices associated with states of a quantum system described in a *d*-dimensional Hilbert space. A fundamental basis for qubits ($\dim {\mathsf {H}}=2$) is formed by the three Pauli matrices and the $2\times 2$ identity matrix. In this case, any density matrix can be represented by a three-dimensional vector, the *Bloch vector*, that lies within the unit ball in ${\mathbb {R}}^3$ whose boundary is the *Bloch sphere*. The points on the spherical surface are in bijective correspondence with the pure states. In higher dimensions, the set of quantum states is a convex body with a much more complicated geometry and it is no longer simply represented as a unit ball. In general, for any *j*, *k*, *l* such that $1\le j\le d^2-1$, $0\le k<l\le d-1$, the *generalized Pauli matrices* on ${\mathbb {C}}^d$ can be defined as follows^4^:

1 $$\begin{aligned} \sigma _j=: \left\{ \begin{array}{ll} \left \vert {\frac{k}{d-1}} \right \rangle \left \langle {\frac{l}{d-1}}\right \vert +\left \vert {\frac{l}{d-1}} \right \rangle \left \langle {\frac{k}{d-1}}\right \vert &{}\quad {\text { if }}\,j\le \frac{d(d-1)}{2}\,\hbox { and }\,j=\frac{k(1-k)}{2}+(d-2) k+l;\\ \\ -i\left \vert {\frac{k}{d-1}} \right \rangle \left \langle {\frac{l}{d-1}}\right \vert +i\left \vert {\frac{l}{d-1}} \right \rangle \left \langle {\frac{k}{d-1}}\right \vert &{} \quad {\text { if }}\,\frac{d(d-1)}{2}< j\le d(d-1)\,\hbox { and }\, j=\frac{d(d-1)+k(1-k)}{2}+(d-2) k+l;\\ \\ \sqrt{\frac{2}{l (l+1)}}\left ({\sum }_{k=0}^{l-1}\left \vert {\frac{k}{d-1}} \right \rangle \left \langle {\frac{k}{d-1}}\right \vert -l\left \vert {\frac{l}{d-1}} \right \rangle \left \langle {\frac{l}{d-1}}\right \vert \right )&{} \quad {\text { if }}\,j>d(d-1)\,\hbox { and }\,j=d(d-1)+l; \end{array} \right. \end{aligned}$$

where $\left\{ \big \vert {\frac{k}{d-1}} \big \rangle \right\} _{k=0,\ldots ,d-1}$ denotes the canonical basis of ${\mathbb {C}}^d$. The generalized Pauli matrices $\{\sigma _j\}_{j=1,\ldots ,d^2-1}$ are the standard generators of the special unitary group *SU*(*d*). Together with the $d\times d$ identity matrix $\mathtt {I}_d$, the generalized Pauli matrices form an orthogonal (the orthogonality is with respect to the Hilbert–Schmidt product $(A,B)_{HS}={\mathrm{tr}}(A^\dagger B)$) basis of the real space of $d\times d$ Hermitian matrices. Let $\rho$ be a density operator on ${\mathbb {C}}^d$, the expansion of $\rho$ with respect to the orthogonal basis $\{\mathtt {I}_d,\sigma _j:1\le j\le d^2-1\}$ is:

2 $$\begin{aligned} \rho =\frac{1}{d}\Big (\mathtt {I}_d+\sqrt{\frac{d(d-1)}{2}}\sum _{j=1}^{d^2-1}b^{(\rho )}_j\sigma _j\Big ), \end{aligned}$$

where $b^{(\rho )}_j=\sqrt{\frac{d}{2(d-1)}}{\mathrm{tr}}(\rho \,\sigma _j)\in {\mathbb {R}}$. The coordinates ${\mathbf{b}}^{(\rho )}=(b^{(\rho )}_1,\ldots ,b^{(\rho )}_{d^2-1})$ represent the Bloch vector associated to $\rho$ with respect to the basis $\{\mathtt {I}_d,\sigma _j:1\le j\le d^2-1\}$, which lies within the hypersphere of radius 1. For $d>2$, the points contained in the unit hypersphere of ${\mathbb {R}}^{d^2-1}$ are not in bijective correspondence with quantum states on ${\mathbb {C}}^d$ such as in the case of a single qubit. However, any vector within the closed ball of radius $\frac{2}{d}$ gives rise to a density operator^5^.

A complex vector can be encoded into a pure state in the following way:

3 $$\begin{aligned} {\mathbb {C}}^n\ni {\mathbf{x}}\mapsto \big \vert {\mathbf{x}} \big \rangle = \frac{1}{\sqrt{\parallel {\mathbf{x}}\parallel ^2+1}}\left( \sum _{\alpha =0}^{n-1}x_\alpha \big \vert {\alpha } \big \rangle + \big \vert {n} \big \rangle \right) \in {\mathsf {H}}, \end{aligned}$$

where $\{\big \vert {\alpha } \big \rangle \}_{\alpha =0,\ldots ,n}$ is the computational basis of the $(n+1)$-dimensional Hilbert space ${\mathsf {H}}$, identified as the standard basis of ${\mathbb {C}}^{n+1}$. The map defined in (3), called *amplitude encoding*, encodes ${\mathbf{x}}$ into the density matrix $\rho _{\mathbf{x}}=\big \vert {\mathbf{x}} \big \rangle \big \langle {\mathbf{x}}\big \vert$ where the additional component of $\big \vert {\mathbf{x}} \big \rangle$ stores the norm of ${\mathbf{x}}$. Nevertheless the quantum encoding ${\mathbf{x}}\mapsto \rho _{\mathbf{x}}$ can be realized in terms of the Bloch vectors ${\mathbf{x}}\mapsto {\mathbf{b}}^{(\rho _{\mathbf{x}})}$. As shown in “Geometric approach to quantum-inspired classifications” section, encoding data into Bloch vectors is useful for saving space resources. The improvement of memory occupation within the Bloch representation is evident when we consider multiple copies of quantum states as tensor products to enlarge the dimension of the representation space (kernel trick). For instance, given two copies of a density operator $\rho _{\mathbf{x}}\otimes \rho _{\mathbf{x}}$ on ${\mathbb {C}}^3\otimes {\mathbb {C}}^3$ (encoding a real feature vector ${\mathbf{x}}\in {\mathbb {R}}^2$), instead of using a matrix of 81 real elements one can store a vector of just 20 entries obtained deleting redundant and null components from the Bloch vector.

## Quantum-inspired classifiers

In this section we introduce the quantum-inspired classifiers that we consider in the present work. The classifier based on Helstrom state discrimination^3,6^ and some nearest mean classifiers based on operator distances among density matrices encoding data. Let us focus on the case of binary classification of *n*-dimensional complex feature vectors, the Helstrom classifier (or Helstrom Quantum Centroid) is based on the following three ingredients: (1) a quantum encoding of the feature vectors into density operators ${\mathbb {C}}^n\ni {\mathbf{x}}\mapsto \rho _{\mathbf{x}}\in {\mathfrak {S}}({\mathsf {H}})$; (2) the construction of the quantum centroids of the two classes $C_1$ and $C_2$ of training points:

4 $$\begin{aligned} \rho _{i}:=\frac{1}{|C_{i}|}\sum _{{\mathbf{x}}\in C_{i}} \rho _{\mathbf{x}}\quad i=1,2 ; \end{aligned}$$

(3) application of the Helstrom discrimination on the two quantum centroids in order to assign a label to a new data instance.

Let us briefly introduce the notion of quantum state discrimination. Given a set of arbitrary quantum states with respective a priori probabilities $R=\{(\rho _1, p_1),\ldots ,(\rho _N, p_N)\}$, in general there is no a measurement process that discriminates the states without errors. More formally, there does not exist a POVM, i.e. a collection $E=\{E_i\}_{i=1,\ldots ,N}\subset {\mathscr {B}}^+({\mathsf {H}})$ such that $\sum _{i=1}^N E_i =\mathtt {I}$, satisfying the following property: ${\mathrm{tr}}(E_i\rho _j)=0$ when $i\not =j$ for all $i,j=1,\ldots ,N$. The probability of a successful state discrimination of the states in *R* performing the measurement *E* is:

5 $$\begin{aligned} {\mathbb {P}}_E(R)=\sum _{i=1}^N p_i {\mathrm{tr}}(E_i \rho _i). \end{aligned}$$

An interesting and useful task is finding the optimal measurement that maximizes the probability (5). Helstrom provided a complete characterization of the optimal measurement $E_{opt}$ for $R=\{(\rho _1,p_1), (\rho _2,p_2)\}$^2^. $E_{opt}$ can be constructed as follows. Let $\Lambda :$ = $p_1\rho _1-p_2\rho _2$ be the *Helstrom observable* whose positive and negative eigenvalues are, respectively, collected in the sets $D_+$ and $D_-$. Consider the two orthogonal projectors:

6 $$\begin{aligned} P_\pm :=\sum _{\lambda \in D_{\pm}} P_\lambda , \end{aligned}$$

where $P_\lambda$ projects onto the eigenspace of $\lambda$. The measurement $E_{opt}:$ $=\{P_+, P_-\}$ maximizes the probability (5) that attains the *Helstrom bound* $h_b(\rho _1,\rho _2)=p_1{\mathrm{tr}}(P_+\rho _1)+p_2{\mathrm{tr}}(P_-\rho _2)$.

Helstrom quantum state discrimination can be used to implement a binary classifier^6^. Let $\{({\mathbf{x}}_1, y_1),\ldots ,({\mathbf{x}}_M, y_M)\}$ be a training set with $y_i\in \{1,2\}$ $\forall i=1,\ldots ,M$. Once a quantum encoding ${\mathbb {C}}^n\ni {\mathbf{x}}\mapsto \rho _{\mathbf{x}}\in {\mathfrak {S}}({\mathsf {H}})$ has been selected, one can construct the quantum centroids $\rho _1$ and $\rho _2$ as in (23) of the two classes $C_{1,2}=\{{\mathbf{x}}_i: y_i=1,2\}$. Let $\{P_+, P_-\}$ be the Helstrom measurement defined by the set $R=\{(\rho _1,p_1),(\rho _2,p_2)\}$, where the probabilities attached to the centroids are $p_{1,2}=\frac{|C_{1,2}|}{|C_1|+|C_2|}$. The *Helstrom classifier* applies the optimal measurement for the discrimination of the two quantum centroids to assign the label *y* to a new data instance ${\mathbf{x}}$, encoded into the state $\rho _{\mathbf{x}}$, as follows:

7 $$\begin{aligned} y({\mathbf{x}})=\left\{ \begin{array}{ll} 1 &{}\quad \text{ if } \, {\mathrm{tr}}(P_+\rho _{\mathbf{x}})\ge {\mathrm{tr}}(P_-\rho _{\mathbf{x}})\\ 2 &{}\quad \text{ otherwise } \end{array} \right. \end{aligned}$$

A strategy to increase the accuracy in classification is given by the construction of the tensor product of *k* copies of the quantum centroids $\rho _{1,2}^{\otimes k}$ enlarging the Hilbert space where data are encoded. The corresponding Helstrom measurement is $\{P_+^{\otimes k}, P_-^{\otimes k}\}$, and the Helstrom bound satisfies^6^:

8 $$\begin{aligned} h_b(\rho _1^{\otimes k}, \rho _2^{\otimes k})\le h_b\left( \rho _1^{\otimes (k+1)}, \rho _2^{\otimes (k+1)}\right) \quad \forall k\in {\mathbb {N}}. \end{aligned}$$

Enlarging the Hilbert space of the quantum encoding, one increases the Helstrom bound obtaining a more accurate classifier. Since the Helstrom classifier is similar to a support vector machine with linear kernel^7^, considering many copies of the encoding quantum states give rise to a kernel trick. The corresponding computational cost is evident; however, in the following, we observe that in the case of real input vectors, the space can be enlarged saving time and space by means of the encoding into Bloch vectors.

Generally speaking, quantum state discrimination approaches consider global measurements or local measurements with classical feed-forward^8^. Unambiguous state discrimination requires more measurement outcomes than the dimension of the Hilbert space, the measurement takes the form of a POVM and identifies the state with certainty or gives an inconclusive outcome. States must have non-overlapping supports (i.e. the space spanned by the eigenvectors with non-zero eigenvalues for each state must not overlap with that of any other state in the ensemble). Maximum confidence sometimes yields incorrect answers^9^. Contrary, the minimum-error measurement strategy is to correctly identify the state as often as possible. For minimum error and unambiguous discrimination, optimization can be treated as a semi-definite program and particular instances can be solved efficiently numerically .

Helstrom provided an analytic closed-form solution for two states with the minimum probability of error and arbitrary prior probabilities. The square-root measurement, also known as *Pretty Good measurement*, defined by:

9 $$\begin{aligned} E_i=p_i\rho ^{-\frac{1}{2}}\rho _i\sqrt{\rho }^{-\frac{1}{2}}, \end{aligned}$$

where $\rho =\sum _i p_i\rho _i$, is the optimal minimum-error when states satisfy certain symmetry properties^10^. Clearly to distinguish between *n* centroids we need a measurement with at most *n* outcomes. It is sometimes optimal to avoid measurement and simply guess that the state is the a priori most likely state.

The optimal POVM $\{E_i\}_i$ for minimum-error state discrimination over $R=\{(\rho _1, p_1),\ldots ,(\rho _N, p_N)\}$ satisfies the following necessary and sufficient Helstrom conditions^11^:

10 $$\begin{aligned} \Gamma -p_i\rho _i \ge 0\quad \forall i, \end{aligned}$$

where the Hermitian operator, also known as *Lagrange operator*, is defined by $\Gamma :=\sum _i p_i\rho _i\, E_i$. It is also useful to consider the following properties which can be obtained from the above conditions:

11 $$\begin{aligned} E_j (p_j\rho _j -p_i\rho _i) E_i =0\quad \forall i,j . \end{aligned}$$

For each *i* the operator $\Gamma -p_i\rho _i$ can have two, one, or no zero eigenvalues, corresponding to the zero operator, a rank-one operator, and a positive-definite operator, respectively. In the first case, we use the measurement $\{E_i=\mathtt {I}, E_{i \not = j}=0\}$ for some *i* where $p_i \ge p_j$ $\forall j$, i.e. the state belongs to the a priori most likely class. In the second case, if $E_i\not = 0$, it is a weighted projector onto the corresponding eigenstate. In the latter case, it follows that $E_i=0$ for every optimal measurement.

Given the following Bloch representations:

12 $$\begin{aligned} \Gamma =\frac{1}{d}\Big (a\,\mathtt {I}_d+\sqrt{\frac{d(d-1)}{2}}\sum _{j=1}^{d^2-1}b^{}_j\sigma _j\Big )\,\, ,\quad \rho _i=\frac{1}{d}\Big (\mathtt {I}_d+\sqrt{\frac{d(d-1)}{2}}\sum _{j=1}^{d^2-1}b^{(i)}_j\sigma _j\Big )\, \, , \end{aligned}$$

in order to determine the Lagrange operator in ${\mathbb {C}}^d$ we need $d^2$ independent linear constraints:

13 $$\begin{aligned} 2 p_i \Big (a-{\widehat{{\mathbf{b}}}}^{(i)}\cdot {\mathbf{b}}-\frac{p_i}{2}(1-|{\widehat{{\mathbf{b}}}}^{(i)}|^2)\Big )=a^2-|{\mathbf{b}}|^2. \end{aligned}$$

A measurement with more than $d^2$ outcomes can always be decomposed as a probabilistic mixture of measurements with at most $d^2$ outcomes. Therefore, if the number of classes is greater than or equal to $d^2$ and we get $d^2$ linearly independent equations, we construct the Lagrange operator and derive the optimal measurements. From the geometric point of view, we obtain the unit vectors corresponding to the rank-1 projectors $E_i=\frac{1}{d}\Big (\mathtt {I}_d+\sqrt{\frac{d(d-1)}{2}}\sum _{j=1}^{d^2-1}n^{(i)}_j\sigma _j\Big )$:

14 $$\begin{aligned} \mathbf{n }^{(i)}=\frac{{\widehat{{\mathbf{b}}}}^{(i)}-a{\mathbf{b}}}{|{\widehat{{\mathbf{b}}}}^{(i)}-a{\mathbf{b}}|}. \end{aligned}$$

It is also possible to further partition the classes in order to increase the number of centroids and of the corresponding equations. An unlabelled point ${{\widehat{{\mathbf{x}}}}}$ is associated with the first label *y* such that ${\mathbf{b}}^{({\hat{{\mathbf{x}}}})}\cdot \mathbf{n }^{(y)}=\max _i {\mathbf{b}}^{({\hat{{\mathbf{x}}}})}\cdot \mathbf{n }^{(i)}$, where $d=\lceil \sqrt{length({\mathbf{x}})+2}\rceil$. Such a geometric construction of the minimum-error state discrimination will be tested over a case-study of medical relevance as reported in “Method and experimental results” section.

The quantum-inspired nearest mean classifiers that we consider in this paper are essentially based on the following general observation: once encoded data into density matrices one can use an operator distance, suitable for quantum state distinguishability, to perform nearest mean classification. In^1^, the authors consider the trace distance that can be computed in terms of Euclidean distance among Bloch vectors. Here we focus on the Bures distance, the Hellinger distance and the Jensen–Shannon distance respectively defined as:

15 $$\begin{aligned} d_{B}(\rho _1,\rho _2)= & {} \sqrt{2-2\,{\mathrm{tr}}\sqrt{\sqrt{\rho _1}\rho _2\sqrt{\rho _1}}}, \end{aligned}$$

16 $$\begin{aligned} d_{He}(\rho _1,\rho _2)= & {} \sqrt{2-2\,{\mathrm{tr}}(\sqrt{\rho _1}\sqrt{\rho _2})}. \end{aligned}$$

17 $$\begin{aligned} d_{JS}(\rho _1,\rho _2)= & {} -{\mathrm{tr}}\Big (\frac{\rho _1+\rho _2}{2}\log \frac{\rho _1+\rho _2}{2}\Big )+\frac{{\mathrm{tr}}(\rho _1\log \rho _1)+{\mathrm{tr}}(\rho _2\log \rho _2)}{2}. \end{aligned}$$

In the next section we explicitly define the nearest mean classifiers, based on the distances (15), (16), (17), within the data encoding into Bloch vectors of density operators in order to take advantage of the geometric approach.

## Geometric approach to quantum-inspired classifications

In this section we discuss the encoding of real feature vectors into Bloch vectors of density operators in order to perform quantum-inspired classification. In particular we observe how the *Bloch representation* turns out to be a useful tool to reduce memory consumption in defining feature maps into higher dimensional spaces.

Within the quantum encoding (3), a real vector ${\mathbf{x}}\in {\mathbb {R}}^{d-1}$ is encoded in a projector operator $\rho _{{\mathbf{x}}}=\big \vert {\mathbf{x}} \big \rangle \big \langle {\mathbf{x}}\big \vert$, on a *d*-dimensional Hilbert space where $d\ge 2$. For simplicity, we consider an input vector $[x_1,x_2]\in {\mathbb {R}}^2$ and the corresponding projector operator $\rho _{[x_1,x_2]}$ on ${\mathbb {C}}^3$. By easy computations, one can see that the Bloch vector of $\rho _{[x_1,x_2]}$ has null components:

18 $$\begin{aligned} {\mathbf{b}}^{(x_1,x_2)}=\frac{1}{1+x_1^2+x_2^2}\Big [2 x_1 x_2,2 x_1,2 x_2,0,0,0,x_1^2-x_2^2,\frac{x_1^2+x_2^2-2}{\sqrt{3}}\Big ]. \end{aligned}$$

Instead of using a matrix with nine real elements to represent $\rho _{[x_1,x_2]}$, memory occupation can be improved by considering only the non-zero components of the Bloch vector. In general, the technique of removing the components that are zero or repeated several times allows reducing the space and the calculation time considering only the significant values that allow to carry out the classification.

Generally speaking, defining a quantum encoding is equivalent to select a feature map to represent feature vectors into a space of higher dimension. In this sense data representation into quantum states can be considered a way to perform kernel tricks. In the case of the considered quantum encoding ${\mathbb {R}}^2\ni [x_1,x_2]\mapsto \rho _{[x_1,x_2]}\in {\mathfrak {S}}({\mathbb {C}}^3)$, in view of (18) the nonlinear explicit injective function $\varphi :{\mathbb {R}}^2\rightarrow {\mathbb {R}}^5$ to encode data into Bloch vectors can be defined as follows:

19 $$\begin{aligned} \varphi ([x_1,x_2]):=\frac{1}{x_1^2+x_2^2+1}\Big [2 x_1 x_2,2 x_1,2 x_2,x_1^2-x_2^2,\frac{x_1^2+x_2^2-2}{\sqrt{3}}\Big ]. \end{aligned}$$

From a geometric point of view, the mapped feature vectors are indeed points on the surface of a hyper-hemisphere. Within this representation, the centroids for classification can be calculated as:

20 $$\begin{aligned} {\bar{{\mathbf{x}}}}^{(\varphi )}_{i}:=\frac{1}{|C_{i}|}\sum _{{\mathbf{x}}\in C_{i}} \varphi ({\mathbf{x}})\quad i=1,2 . \end{aligned}$$

In general, such centroids are points inside the hypersphere that do not have an inverse image in terms of density operators, however they can be rescaled to a Bloch vector as discussed below.

Data points can also be encoded in a smaller space using the following encoding from ${\mathbb {R}}^2$ to density operators of ${\mathbb {C}}^2$:

21 $$\begin{aligned} \rho _{[x_1,x_2]}=\frac{1}{2}\Big (\mathtt {I}_{2}+\sum _{j=1}^{3}b_j\sigma _j\Big )\in {\mathfrak {S}}({\mathbb {C}}^2), \end{aligned}$$

where the Bloch vector ${\mathbf{b}}=\varphi ([x_1,x_2])\in {\mathbb {R}}^3$ and $\varphi ([x_1,x_2]):=\frac{1}{\sqrt{x_1^2+x_2^2+1}}[x_1,x_2,1]$. In this case, if the quantum centroids are calculated as in (20), they are points inside the Bloch sphere of a qubit then correspond to density operators. As shown below, considering Helstrom classifier, within this quantum encoding it is less accurate than the encoding into ${\mathbb {C}}^3$ as expected by any representation of data in a space of lower dimension.

In order to improve the accuracy of the classification, one can increase the dimension of the representation space providing *k* copies of the quantum states, in terms of a tensor product, encoding data instances and centroids. According to the quantum formalism, multiple copies of the states are described in a tensor product Hilbert space with a strong impact in terms of computational space (from dimension $d-1$ to $d^{2 k}$) and time. Following the geometric approach, considering the significant values that allow to carry out the classification, the explicit function $\varphi :{\mathbb {R}}^2\rightarrow {\mathbb {R}}^{20}$ for two copies of the density operators on ${\mathbb {C}}^3$ can be defined as follows:

$$\begin{aligned} \varphi ([x_1,x_2]):= & {} \frac{1}{(x_1^2+x_2^2+1)^2}\Big [2 x_1^3 x_2,2 x_1^3,2 x_1^2 x_2^2,2 x_1^2 x_2,2 x_1^2,2 x_1 x_2^3,2 x_1 x_2^2,2 x_1 x_2,2 x_1,2 x_2^3,\\&2 x_2^2,2 x_2, x_1^2 (x_1-x_2)(x_1+x_2),\frac{x_1^2(x_1^2+x_2^2-2)}{\sqrt{3}},\frac{x_1^2(x_1^2-2 x_2^2+1)}{\sqrt{6}},\\&\frac{x_1^4-4 x_2^4+x_1^2(2 x_2^2+1)}{\sqrt{10}},\frac{x_1^2+x_1^4-5 x_2^2+2 x_1^2 x_2^2+x_2^4}{\sqrt{15}},\frac{x_1^4+x_2^2+x_2^4+x_1^2(2 x_2^2-5)}{\sqrt{21}},\\&\frac{x_1^4-6 x_2^2+x_2^4+2 x_1^2(x_2^2+1)}{2\sqrt{7}},\frac{1}{6}(x_1^2+x_2^2-2)(x_1^2+x_2^2+4)\Big ]. \end{aligned}$$

In particular, removing null and multiple entries, we consider only 20 values instead of 81 for two copies, 51 values instead of 729 for three copies and so on. However, one must also take into account high-precision numbers and track the propagation of the numerical error.

Consider the quantum amplitude encoding of *d*-dimensional real feature vectors into pure states as introduced in “Quantum encoding” section:

22 $$\begin{aligned} {\mathbb {R}}^{d}\ni {\mathbf{x}}\mapsto \big \vert {{\mathbf{x}}} \big \rangle = \frac{1}{\sqrt{\parallel {\mathbf{x}}\parallel ^2+1}}\left( \sum _{\alpha =0}^{d-1}x_\alpha \big \vert {\alpha } \big \rangle + \big \vert {d} \big \rangle \right) \in {\mathbb {C}}^{d+1}, \end{aligned}$$

where $\{\big \vert {\alpha } \big \rangle \}_{\alpha =0,\ldots ,d}$ is the computational basis of the considered $(d+1)$-dimensional Hilbert space. The quantum centroids of the classes $C_1,\ldots , C_M$ of training points are defined by the mixed states:

23 $$\begin{aligned} \rho _{i}:=\frac{1}{|C_{i}|}\sum _{{\mathbf{x}}\in C_{i}}\rho _{\mathbf{x}}\quad \text{ with }\quad \rho _{\mathbf{x}}=\big \vert {{\mathbf{x}}} \big \rangle \big \langle {{\mathbf{x}}}\big \vert ,\quad i=1,\ldots ,M \quad \text{ and } M \text{ is } \text{ the } \text{ number } \text{ of } \text{ classes }. \end{aligned}$$

Since any density operator $\rho _{\mathbf{x}}$ can be represented in terms of its Bloch vector ${\mathbf{b}}^{({\mathbf{x}})}$, we can adopt the *Bloch representation* of data ${\mathbf{x}}\mapsto {\mathbf{b}}^{({\mathbf{x}})}$ so the centroids can be calculated in terms of Bloch vectors:

24 $$\begin{aligned} {\mathbf{b}}^{(i)}:=\frac{1}{|C_i|}\sum _{{\mathbf{x}}\in C_i} {\mathbf{b}}^{({\mathbf{x}})}, \end{aligned}$$

noting that ${\mathbf{b}}^{(i)}$ does not correspond to the Bloch vector of the quantum centroid $\rho _i$ calculated in (23). In fact, ${\mathbf{b}}^{(i)}$ lies inside the hypersphere in ${\mathbb {R}}^{d^2+2d}$ then it is not necessarily the Bloch vector of a density operator for $d>1$. However it can be contracted into the hypersphere of radius $\frac{2}{d+1}$ to individuate a Bloch vector of a density operator, thus we define the *contracted centroid* ${\widehat{{\mathbf{b}}}}^{(i)}:=\frac{2}{d+1}{\mathbf{b}}^{(i)}$. Obviously, not even ${\widehat{{\mathbf{b}}}}^{(i)}$ is the Bloch vector of the quantum centroid $\rho _i$ however it represents a valid density operator, say ${{\widehat{\rho}}}_{i}$, on ${\mathbb {C}}^{d+1}$ that can be adopted as an alternative definition of centroid.

Given the class $C_i$ of data points, let us list different notions of centroid of $C_i$ that we can define within a fixed quantum encoding ${\mathbf{x}}\mapsto \rho _{\mathbf{x}}$:

1. Quantum centroid $$\rho _{i}:=\frac{1}{|C_{i}|}\sum _{{\mathbf{x}}\in C_{i}}\rho _{\mathbf{x}}$$;
2. Quantum encoding $\rho _{{\overline{{\mathbf{x}}}}_i}$ of the classical centroid ${\overline{{\mathbf{x}}}}_i:=\frac{1}{|C_{i}|}\sum _{{\mathbf{x}}\in C_{i}}{\mathbf{x}}$;
3. Mean of the Bloch vectors ${\mathbf{b}}^{(i)}:=\frac{1}{|C_i|}\sum _{{\mathbf{x}}\in C_i} {\mathbf{b}}^{({\mathbf{x}})}$;
4. Contracted centroid ${\widehat{{\mathbf{b}}}}^{(i)}:=\frac{2}{d+1}{\mathbf{b}}^{(i)}$ that is a Bloch vector itself.

In general, we have that $\rho _i\not =\rho _{{\overline{{\mathbf{x}}}}_i}$ and ${\widehat{{\mathbf{b}}}}^{(i)}$ is not the Bloch vector of $\rho _i$ or $\rho _{{\overline{{\mathbf{x}}}}_i}$. In the construction of the nearest mean classifiers with operator distances we choose ${\widehat{{\mathbf{b}}}}^{(i)}$ as definition of centroid in order to select the encoding that is less memory consuming and to represent centroids by quantum states so that the calculation of the considered operator distances is meaningful in terms of distinguishability of quantum states.

Let us consider a binary classification problem (the multi-class generalization is straightforward). As suggested in^7^, we can define a classification algorithm that evaluates the Bures distance between the pure state encoding a test point and the centroids that correspond to mixed states. The fidelity between density operators, defined as ${\mathscr {F}}(\rho _1,\rho _2)=\left( {\mathrm{tr}}\sqrt{\sqrt{\rho _1}\rho _2\sqrt{\rho _1}}\right) ^2$, reduces to ${\mathscr {F}}(\rho _1,\rho _2)=\langle \psi _1|\rho _2|\psi _1\rangle$ when $\rho _1=\big \vert {\psi _1} \big \rangle \big \langle {\psi _1}\big \vert$. Therefore the Bures distance between the pure state $\rho _1$ and the generic state $\rho _2$ can be expressed in term of the Bloch representation as follows:

25 $$\begin{aligned} d_B(\rho _1, \rho _2)=\sqrt{2-2\sqrt{\frac{1}{n}\left( 1+(n-1) {\mathbf{b}}^{(1)}\cdot {\mathbf{b}}^{(2)}\right)}}\equiv D_B\left( {\mathbf{b}}^{(1)}, {\mathbf{b}}^{(2)}\right) , \end{aligned}$$

where ${\mathbf{b}}^{(1)}$ and ${\mathbf{b}}^{(2)}$ are the Bloch vectors of $\rho _1$ and $\rho _2$ respectively and *n* is the dimension of the Hilbert space of the quantum encoding. The formula (25) can be directly derived from

26 $$\begin{aligned} {\mathrm{tr}}(\rho _1\rho _2)=\frac{1}{n}(1+(n-1){\mathbf{b}}^{(1)}\cdot {\mathbf{b}}^{(2)}), \end{aligned}$$

that is an immediate consequence of the fact that the generalized Pauli matrices are traceless and satisfy ${\mathrm{tr}}(\sigma _i\sigma _j)=2\delta _{ij}$. Thus a quantum-inspired nearest mean classifier based on Bures distance for binary classification can be defined by Algorithm 1.
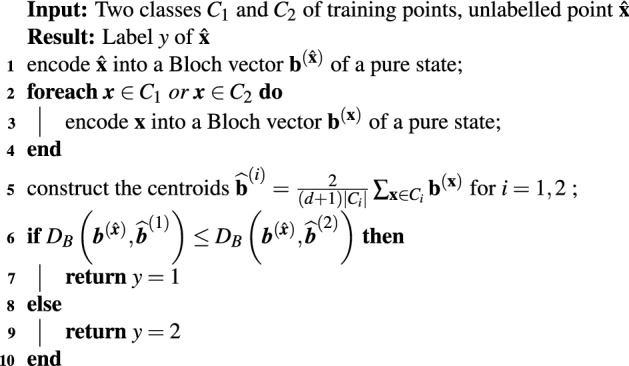
**Algorithm 1**: Quantum-inspired nearest mean classifier based on Bures distance.

Now let us consider the Hellinger distance (16). Assuming that $\rho _1$ is a pure state in a *n*-dimensional Hilbert space, so $\sqrt{\rho _1}=\rho _1$, then the distance can be written as:

27 $$\begin{aligned} d_{He}(\rho _1,\rho _2)=\sqrt{2-2Tr(\rho _1\sqrt{\rho }_2)}=\sqrt{2-\frac{2}{n}(1+(n-1){\mathbf{b}}^{(1)}\cdot \mathbf{d }^{(2)})}\equiv D_{He}({\mathbf{b}}^{(1)},\mathbf{d }^{(2)}), \end{aligned}$$

where ${\mathbf{b}}^{(1)}$ is the Bloch vector of the state $\rho _1$ and $\mathbf{d }^{(2)}$ is the Bloch vector of the operator $\sqrt{\rho _2}$. Therefore a nearest mean classifier based on Hellinger distance, within the Bloch representation, can be defined by Algorithm 2 which provides the square roots of the density operators corresponding to the centroids. A standard calculation is done solving the corresponding eigenvalue problem. Given a density operator, let *Diag* be the function returning a unitary matrix *U* and a diagonal matrix $\Lambda$ such that $\rho =U\Lambda U^{-1}$. Obviously $\sqrt{\rho }=U\sqrt{\Lambda }U^{-1}$ where $\sqrt{\Lambda }$ is the diagonal matrix given by the square roots of the eigenvalues of $\rho$.

In Algorithm 2, the function *BlochVector* returns the Bloch vector of a given density operator and $BlochVector^{-1}$ is its inverse. On the one hand, the centroids are computed in terms of Bloch vectors but they are translated into operators to compute the Hellinger distance. On the other hand the training points are processed directly in terms of their Bloch representation.

In the case of feature vectors in ${\mathbb {R}}^2$, quantum-inspired classification can also be applied in a smaller space than ${\mathbb {C}}^3$ using the encoding (21). In other words, data points are encoded into Bloch vectors of pure states of a single qubit, so a centroid calculated as in (24) is a vector inside the Bloch sphere then always represents a quantum state $\rho _i$. In this low-dimensional case, Hellinger distance and Jensen–Shannon distance between dataset elements and the centroids can be calculated with the following simplified formulas:

28 $$\begin{aligned} d_{He}(\rho _{\mathbf{x}},\rho _i)= & {} \sqrt{2-\sqrt{2}\frac{1+\sqrt{1-r^2}+{\mathbf{b}}^{({\mathbf{x}})}\cdot {\mathbf{b}}^{(i)}}{\sqrt{1-r}+\sqrt{1+r}}}, \end{aligned}$$

29 $$\begin{aligned} d_{JS}(\rho _{\mathbf{x}},\rho _i)= & {} \frac{1-r}{4} \log \frac{1-r}{2}+\frac{1+r}{4} \log \frac{1+r}{2}+\frac{2-s}{4} \log \frac{2-s}{4}+\frac{2+s}{4} \log \frac{2+s}{4}, \end{aligned}$$

where $r=|{\mathbf{b}}^{(i)}|$ and $s=|{\mathbf{b}}^{({\mathbf{x}})}+{\mathbf{b}}^{(i)}|$. Thus the corresponding near mean classifiers can be defined by Algorithm 1 with (28) and (29) in place of the Bures distance.
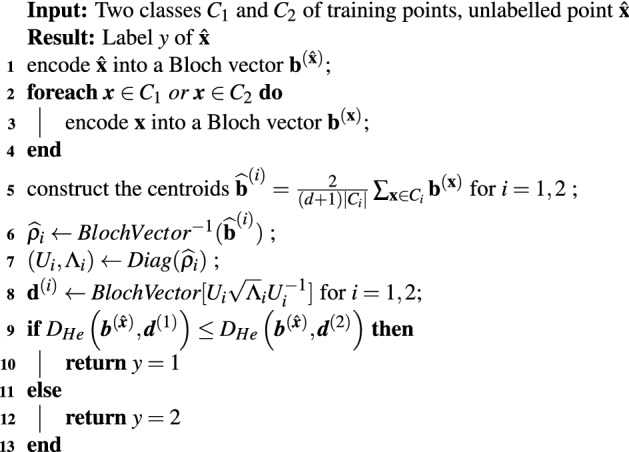
**Algorithm 2**: Quantum-inspired nearest mean classifier based on Hellinger distance.

## Method and experimental results

In this section, we present some numerical results obtained by the implementation of the Helstrom classifier and the considered quantum-inspired nearest mean classifiers compared to classical algorithms. We run the Helstrom classifier and the nearest mean classifiers with several distances (Euclidean, Bures, Hellinger, Jensen–Shannon) compared to the following well-known classifiers that we list with respective parameters, settings and main characteristics:

- *K*-*Nearest Neighbors*: number of neighbors $K=3$, Euclidean distance as distance measure, uniform weights in each neighborhood;
- *Gaussian Process*: kernel $1.0*RBF(1.0)$, maximum number of iterations in Newton’s method $=100$;
- *Linear SVM*: regularization parameter $=0.025$, no limit on iterations within solver;
- *RBF SVM*: regularization parameter $=1.0$, kernel coefficient for RBF $\gamma =2$, no limit on iterations within solver;
- *Neural Network (multi-layer perceptron classifier)*: number of hidden layers $=1$, number of neurons in the hidden layer $=100$, activation function $f(x)=\max (0,x)$, L2 penalty parameter $=1$, learning rate $=0.001$, maximum number of epochs in weight optimization $=1000$, weight optimization performed by stochastic gradient;
- *Quadratic Discriminant Analysis*: tolerance for a singular value to be considered significant $=0.0001$.
- *Decision Tree*: maximum depth of the tree $=5$, minimum number of samples required to split an internal node $=2$, Gini impurity for evaluating the quality of a split.
- *Random Forest*: maximum depth $=5$, number of trees in the forest $=10$, minimum number of samples required to split an internal node $=2$, Gini impurity for evaluating the quality of a split.
- *AdaBoost*^12^: Decision Tree as base classifier, maximum number of estimators $=50$, learning rate $=1.0$,
- *Naive Bayes*: Portion of the largest variance of all features that is added to variances for calculation stability $=10^{-9}$;

In order to compare the results with previous papers, we consider the following toy data and benchmark datasets from PMLB public repository^13^: *moons, cicles, linearly separable, analcatdata aids, analcatdata asbestos, analcatdata boxing2, Hill Valley with noise, Hill Valley without noise, lupus, prnn synth.* For each dataset we randomly select $80\%$ of the data to create a training set and use the residual $20\%$ for the evaluation.

For simplicity, we only consider the first two features of the datasets, i.e., an input vector $[x_1,x_2]\in {\mathbb {R}}^2$ and quantum-inspired classifiers in ${\mathbb {C}}^2$ within the encoding (21). We repeated the same procedure 100 times and calculated the average accuracy in Table 1. The results w.r.t. the *F*1-score are reported in Table 2. Since the Jensen and Hellinger distances generally do not provide better results than the Euclidean and Bures distances, even in the presence of more preparations of the same state, we will consider only the latter (Tables 3, 4, 5, 6, 7).

**Table 1:** Average accuracy with the first 2 features.

Dataset	Helstrom	Euclide	Bures	Hellinger	Jensen	Nearest neighbors	Gaussian process	Linear SVM	RBF SVM	Neural net	QDA	Decision tree	Random Forest	AdaBoost	Naive Bayes
Moons	0.529	0.842	0.8445	0.8425	0.842	0.952	0.9365	0.8325	0.944	0.844	0.834	0.894	0.9035	0.9135	0.8385
Cicles	0.4855	0.631	0.509	0.5555	0.6525	0.854	0.8895	0.4065	0.8905	0.8765	0.853	0.835	0.8375	0.8285	0.86
Linearly separable	0.929	0.933	0.936	0.935	0.933	0.9425	0.93	0.9285	0.942	0.939	0.93	0.9065	0.9125	0.896	0.936
Analcatdata aids	0.382	0.31	0.312	0.306	0.308	0.262	0.103	0.386	0.095	0.261	0.252	0.093	0.105	0.205	0.279
Analcatdata asbestos	0.606471	0.714706	0.725882	0.720588	0.714118	0.722941	0.744706	0.695294	0.721176	0.748235	0.728824	0.748235	0.755294	0.695882	0.713529
Analcatdata boxing2	0.548889	0.524815	0.547778	0.536667	0.531111	0.450741	0.521111	0.532222	0.494444	0.522593	0.528519	0.434815	0.44037	0.455926	0.539259
Hill valley with noise	0.481317	0.499835	0.502634	0.504938	0.50465	0.497531	0.516872	0.478189	0.517654	0.488971	0.499383	0.51535	0.509012	0.51465	0.489547
Hill valley without noise	0.489712	0.514486	0.516049	0.508066	0.509259	0.503909	0.49679	0.492222	0.493868	0.501193	0.513827	0.503292	0.505391	0.518189	0.507942
Lupus	0.773333	0.735	0.733333	0.733333	0.734444	0.706111	0.757222	0.756667	0.722222	0.753333	0.742778	0.707778	0.721667	0.665556	0.717778
Prnn synth	0.455	0.8566	0.832	0.8506	0.8558	0.854	0.8622	0.8362	0.868	0.8516	0.8424	0.8232	0.8468	0.8298	0.8362

The best result for each dataset is marked in bold.

**Table 2:** *F*1-score with the first 2 features.

Dataset	Helstrom	Euclide	Bures	Hellinger	Jensen	Nearest neighbors	Gaussian process	Linear SVM	RBF SVM	Neural net	QDA	Decision tree	Random forest	AdaBoost	Naive Bayes
Moons	0.464922	0.840323	0.841519	0.84047	0.840323	0.953685	0.936021	0.829191	0.946382	0.841357	0.83168	0.892442	0.901073	0.91238	0.837082
Cicles	0.629838	0.646017	0.667201	0.680357	0.700705	0.855677	0.886392	0.30833	0.887022	0.873499	0.843021	0.829894	0.833722	0.82728	0.850513
Linearly separable	0.925752	0.927311	0.930675	0.929727	0.927311	0.94043	0.928188	0.92364	0.939444	0.935823	0.92697	0.904343	0.908898	0.894871	0.931908
Analcatdata aids	0.312119	0.290014	0.290706	0.286587	0.28699	0.230239	0.111767	0.360506	0.101949	0.25471	0.252926	0.039859	0.093086	0.191513	0.263386
Analcatdata asbestos	0.423265	0.678411	0.703593	0.689334	0.678303	0.653684	0.686772	0.652546	0.644262	0.696681	0.686944	0.689379	0.696547	0.635218	0.67861
Analcatdata boxing2	0.683352	0.597178	0.637601	0.617339	0.606975	0.491697	0.607008	0.649924	0.568273	0.60897	0.615534	0.474085	0.507492	0.530448	0.630684
Hill valley with noise	0.348074	0.337724	0.291553	0.317597	0.327122	0.498334	0.393383	0.306991	0.372891	0.366183	0.380321	0.453604	0.47774	0.444733	0.430896
Hill valley without noise	0.555846	0.617317	0.636632	0.624886	0.621875	0.512918	0.531402	0.585316	0.604029	0.591217	0.660424	0.506052	0.526377	0.525226	0.642497
Lupus	0.602344	0.669221	0.666688	0.666656	0.667856	0.579922	0.656767	0.582944	0.547894	0.647052	0.630765	0.562144	0.591334	0.546564	0.600368
Prnn synth	0.458623	0.858884	0.840259	0.855394	0.858714	0.851627	0.862972	0.841066	0.868374	0.852702	0.845555	0.819956	0.845004	0.829847	0.838924

**Table 3:** Average accuracy with 2 features mapped into high-dimensional feature space ${\mathbb {R}}^5$.

Dataset	Helstrom	Euclide	Bures	Nearest neighbors	Gaussian process	Linear SVM	RBF SVM	Neural net	QDA	Decision tree	Random Forest	AdaBoost	Naive Bayes
Moons	0.761	0.8355	0.838	0.9365	0.928	0.7995	0.931	0.907	0.9235	0.8055	0.8385	0.788	0.819
Cicles	0.757	0.805	0.7805	0.8315	0.8805	0.4635	0.855	0.886	0.872	0.845	0.8685	0.8565	0.8945
Linearly separable	0.831	0.9325	0.7525	0.948	0.9275	0.921	0.938	0.9355	0.938	0.92	0.9155	0.9055	0.941
Analcatdata aids	0.25	0.236	0.232	0.172	0.089	0.335	0.099	0.217	0.153	0.09	0.11	0.085	0.214
Analcatdata asbestos	0.761765	0.732941	0.708824	0.724118	0.738235	0.612941	0.732941	0.747647	0.6	0.741176	0.753529	0.751176	0.73
Analcatdata boxing2	0.541111	0.518148	0.537037	0.45963	0.507778	0.536667	0.487407	0.516296	0.479259	0.433333	0.445185	0.435926	0.516296
Hill valley with noise	0.483128	0.495226	0.490535	0.494938	0.511852	0.477325	0.527407	0.48856	0.492593	0.512428	0.505926	0.503169	0.487449
Hill valley without noise	0.501852	0.509547	0.138148	0.505761	0.496132	0.486049	0.516749	0.500905	0.54284	0.607078	0.591975	0.58214	0.511646
Lupus	0.737778	0.715	0.720556	0.73	0.745556	0.619444	0.749444	0.746667	0.747222	0.681667	0.704444	0.657222	0.696111
Prnn synth	0.8084	0.8438	0.8386	0.8588	0.8678	0.8466	0.8576	0.857	0.8576	0.8182	0.8346	0.8042	0.8404

**Table 4:** Average accuracy with 2 features mapped into high-dimensional feature space ${\mathbb {R}}^{20}$.

Dataset	Helstrom	Euclide	Bures	Nearest neighbors	Gaussian process	Linear SVM	RBF SVM	Neural net	QDA	Decision tree	Random Forest	AdaBoost	Naive Bayes
Moons	0.8395	0.882	0.8835	0.9495	0.928	0.479	0.933	0.932	0.9175	0.889	0.7975	0.8915	0.8025
Cicles	0.628	0.841	0.846	0.847	0.878	0.4585	0.872	0.8785	0.857	0.832	0.8565	0.845	0.8675
Linearly separable	0.9045	0.9095	0.9165	0.935	0.9375	0.489	0.9415	0.9205	0.9435	0.91	0.87	0.9145	0.918
Analcatdata aids	0.172	0.191	0.187	0.173	0.181	0.327	0.093	0.198	0.318	0.093	0.105	0.084	0.189
Analcatdata asbestos	0.725882	0.724706	0.715882	0.722941	0.732353	0.561176	0.731176	0.738824	0.665294	0.728824	0.743529	0.698235	0.682941
Analcatdata boxing2	0.515926	0.50037	0.522963	0.467407	0.488148	0.536667	0.486667	0.506667	0.492963	0.429259	0.435556	0.445556	0.497407
Hill valley with noise	0.483333	0.488807	0.495556	0.496008	0.51786	0.479053	0.529383	0.492016	0.500165	0.510206	0.504198	0.499671	0.493333
Hill valley without noise	0.496626	0.508148	0.506461	0.505226	0.49284	0.481728	0.518025	0.500247	0.551564	0.561687	0.531399	0.58037	0.505432
Lupus	0.773333	0.715	0.71	0.720556	0.747778	0.622222	0.747778	0.745	0.699444	0.693333	0.68	0.68	0.693889
Prnn synth	0.8466	0.8564	0.8594	0.8622	0.8674	0.7806	0.863	0.873	0.8478	0.8342	0.816	0.833	0.7902

**Table 5:** Average accuracy with 2 features mapped into high-dimensional feature space ${\mathbb {R}}^{81}$.

Dataset	Helstrom	Euclide	Bures	Nearest neighbors	Gaussian process	Linear SVM	RBF SVM	Neural net	QDA	Decision tree	Random Forest	AdaBoost	Naive Bayes
Moons	0.9165	0.903	0.9105	0.945	0.93	0.4805	0.9345	0.9355	0.9	0.871	0.728	0.8985	0.7675
Cicles	0.7605	0.855	0.8785	0.851	0.881	0.453	0.8745	0.8805	0.853	0.822	0.8545	0.829	0.88
Linearly separable	0.9325	0.8765	0.8875	0.938	0.949	0.483	0.9295	0.9195	0.9375	0.935	0.8425	0.9405	0.8315
Analcatdata aids	0.121	0.176	0.173	0.183	0.284	0.341	0.148	0.29	0.084	0.095	0.117	0.084	0.195
Analcatdata asbestos	0.731765	0.715294	0.714118	0.723529	0.72	0.557647	0.732941	0.733529	0.6	0.742353	0.745882	0.747647	0.661176
Analcatdata boxing2	0.51	0.49	0.508889	0.467037	0.49963	0.536667	0.494074	0.507037	0.495185	0.427037	0.442222	0.436667	0.496667
Hill valley with noise	0.483251	0.487984	0.493457	0.500206	0.510905	0.479053	0.529218	0.485885	0.506502	0.508642	0.500206	0.493169	0.498025
Hill valley without noise	0.498477	0.507984	0.509342	0.504609	0.493992	0.483621	0.520082	0.497119	0.562593	0.520082	0.508601	0.546132	0.50963
Lupus	0.773333	0.703333	0.695556	0.701667	0.746667	0.622222	0.748333	0.745556	0.636111	0.678889	0.659444	0.668889	0.639444
Prnn synth	0.8538	0.8546	0.8628	0.857	0.8668	0.488	0.8708	0.8756	0.8552	0.8372	0.7952	0.8344	0.7462

**Table 6:** Average accuracy with 2 features mapped into high-dimensional feature space ${\mathbb {R}}^{122}$.

Dataset	Helstrom	Euclide	Bures	Nearest neighbors	Gaussian process	Linear SVM	RBF SVM	Neural net	QDA	Decision tree	Random Forest	AdaBoost	Naive Bayes
Moons	0.92	0.8915	0.909	0.9375	0.931	0.4665	0.9265	0.9375	0.88	0.859	0.682	0.896	0.6465
Cicles	0.807	0.8445	0.887	0.8525	0.882	0.445	0.871	0.8855	0.7695	0.823	0.857	0.8555	0.892
Linearly separable	0.9365	0.8375	0.856	0.9005	0.9505	0.4735	0.8925	0.908	0.9135	0.9295	0.7665	0.9225	0.713
Analcatdata aids	0.099	0.205	0.211	0.201	0.257	0.37	0.284	0.348	0.084	0.093	0.121	0.084	0.205
Analcatdata asbestos	0.732353	0.698235	0.708235	0.723529	0.729412	0.553529	0.732353	0.731176	0.737647	0.711176	0.742353	0.727647	0.642353
Analcatdata boxing2	0.502593	0.486296	0.502222	0.467778	0.500741	0.536667	0.495556	0.507037	0.538148	0.43	0.45	0.447778	0.498889
Hill valley with noise	0.484362	0.480988	0.494774	0.499012	0.504979	0.479053	0.531193	0.480082	0.50642	0.515885	0.504321	0.518642	0.495103
Hill valley without noise	0.500494	0.504321	0.503251	0.50428	0.49749	0.483621	0.521029	0.490453	0.569218	0.52572	0.501852	0.53214	0.508889
Lupus	0.772778	0.684444	0.685	0.686667	0.726111	0.622222	0.748333	0.74	0.652222	0.651667	0.578333	0.642778	0.582778
Prnn synth	0.8556	0.8174	0.8602	0.8582	0.8616	0.4834	0.874	0.8772	0.8392	0.8286	0.7712	0.824	0.6968

**Table 7:** *F*1-score with 2 features mapped into high-dimensional feature space ${\mathbb {R}}^{122}$.

Dataset	Helstrom	Euclide	Bures	Nearest neighbors	Gaussian process	Linear SVM	RBF SVM	Neural net	QDA	Decision tree	Random Forest	AdaBoost	Naive Bayes
Moons	0.919654	0.888403	0.90364	0.940338	0.931047	0.355928	0.927139	0.940196	0.874958	0.856571	0.679505	0.893739	0.637247
Cicles	0.78895	0.814057	0.884429	0.848254	0.876753	0.286566	0.856554	0.877363	0.743484	0.816633	0.849931	0.849835	0.884564
Linearly separable	0.9331	0.811657	0.840846	0.898957	0.949107	0.352685	0.887571	0.90338	0.908466	0.928714	0.764568	0.920865	0.681532
Analcatdata aids	0.094768	0.191961	0.197471	0.197468	0.218508	0.291432	0.241194	0.292872	0.072162	0.048727	0.09554	0.083706	0.184656
Analcatdata asbestos	0.646487	0.664024	0.685391	0.648116	0.667969	0.03001	0.678992	0.667294	0.653541	0.643194	0.686401	0.674249	0.589861
Analcatdata boxing2	0.607337	0.508385	0.559737	0.507701	0.612686	0.652648	0.574432	0.612221	0.657725	0.465764	0.525866	0.518559	0.549566
Hill valley with noise	0.385468	0.415765	0.410437	0.504632	0.4108	0.338354	0.385776	0.396176	0.335648	0.292807	0.465071	0.451033	0.326622
Hill valley without noise	0.590686	0.590196	0.622812	0.513252	0.547225	0.545744	0.60283	0.564534	0.666941	0.467788	0.521789	0.524884	0.638644
Lupus	0.632978	0.581279	0.585018	0.54156	0.584137	0	0.582675	0.593176	0.534378	0.527788	0.390944	0.523898	0.460007
Prnn synth	0.856229	0.795313	0.860908	0.856145	0.861114	0.366932	0.87431	0.877672	0.839585	0.823271	0.77101	0.819984	0.70899

To correctly compare quantum-inspired classifiers in ${\mathbb {C}}^3$ with the well-known classifiers it is useful to map two features into a higher dimensional feature space ${\mathbb {R}}^5$ with the following explicit function $\varphi :{\mathbb {R}}^2\rightarrow {\mathbb {R}}^5$:

$$\begin{aligned} \varphi _1([x_1,x_2]):=\frac{2}{x_1^2+x_2^2+1}\left[ x_1 x_2, x_1, x_2,\frac{x_1^2-x_2^2}{2},\frac{x_1^2+ x_2^2-2}{2\sqrt{3}}\right] . \end{aligned}$$

For quantum-inspired classifiers in ${\mathbb {C}}^3\otimes {\mathbb {C}}^3$ with two preparations of the same quantum state it is useful the following explicit function $\varphi _2:{\mathbb {R}}^2\rightarrow {\mathbb {R}}^{20}$:

$$\begin{aligned} \varphi _2([x_1,x_2]):= & {} \frac{2}{(x_1^2+x_2^2+1)^2}\Big [ x_1^3 x_2, x_1^3, x_1^2 x_2^2, x_1^2 x_2,x_1^2,x_1 x_2^3,x_1 x_2^2,x_1 x_2,x_1,x_2^3,x_2^2,x_2,\\&\frac{x_1^4-x_1^2 x_2^2}{2},\frac{x_1^4+x_1^2 x_2^2 -2 x_1^2}{2\sqrt{3}}, \frac{x_1^4+x_1^2 x_2^2+x_1^2-3 x_1^2 x_2^2}{2\sqrt{6}}, \frac{x_1^4+x_1^2-4 x_2^4+2 x_1^2 x_2^2}{2\sqrt{10}},\\&\frac{x_1^4+x_1^2-5 x_2^2+2 x_1^2 x_2^2+x_2^4}{2\sqrt{15}}, \frac{x_1^4-5 x_1^2+x_2^2+2 x_1^2 x_2^2+x_2^4}{2\sqrt{21}},\\&\frac{\frac{1}{2}x_1^4+x_1^2-3 x_2^2+2 x_1^2 x_2^2+\frac{1}{2}x_2^4}{2\sqrt{7}}, \frac{2 x_1^4+x_1^2+x_2^2+ x_1^2 x_2^2+2 x_2^4-4}{6}\Big ]. \end{aligned}$$

The dimension of the feature space can be further increased considering multiple copies of the encoding quantum states as density operators in $({\mathbb {C}}^3)^{\otimes 3}$ and $({\mathbb {C}}^3)^{\otimes 4}$ implementing corresponding feature maps that are respectively given by explicit functions $\varphi _3:{\mathbb {R}}^2\rightarrow {\mathbb {R}}^{81}$ and $\varphi _4:{\mathbb {R}}^2\rightarrow {\mathbb {R}}^{122}$.

In the presented experiments we consider the average accuracy and the *F*1-score (Tables 2 and 7) as figures of merit to test and compare the performances of the quantum-inspired classifiers. However *F*-measures do not take true negative into account then average accuracy is considered better for the performance comparison of the classifier. Certainly, it is possible to compare the performances based on different statistic indices including balanced accuracy, sensitivity, specificity, precision, F-measure, Cohen’s k parameter^3^.

Helstrom classifier has been applied and compared with classical algorithms over the following datasets provided by the Wolfram data repository:

1. Death times of male laryngeal cancer patients: https://doi.org/10.24097/wolfram.61527.data.
2. Locations of cancer cases in North Liverpool, UK, annotated with subject type (case or control) marks: https://datarepository.wolframcloud.com/resources/Sample-Data-Liverpool-Cancer.
  State discrimination using the Pretty Good measurement and the geometric Helstrom state discrimination introduced in “Quantum-inspired classifiers” section have been tested over the dataset:
3. Case-control study of esophageal cancer https://doi.org/10.24097/wolfram.41634.data.

The obtained results are reported in Tables 8 and 9 and discussed in the next section.

**Table 8:** Death/alive laryngeal cancer patients and case-control marks of cancer cases in North Liverpool.

	Helstrom	Linear	RadialBasisFunction	Polynomial	Sigmoid	RandomForest	NaiveBayes	NearestNeighbors	LogisticRegression
LarynxCancer	0.52	0.965	0.928333	0.93	0.888333	0.791111	0.712222	0.747778	0.946667
LiverpoolCancer	0.637017	0.799501	0.799501	0.799501	0.790197	0.769658	0.799001	0.799606	0.799501

**Table 9:** Case-control study of esophageal cancer.

	PrettyGood	GeometricHelstrom	Linear	RadialBasisFunction	Polynomial	Sigmoid	RandomForest	NaiveBayes	NearestNeighbors	LogisticRegression
EsophagealCancer	0.336111	0.4	0.293333	0.238333	0.236667	0.218333	0.493889	0.457222	0.241667	0.347222

## Discussion

The low-dimensional experiments, whose results are reported in Table 1, are performed encoding feature vectors of ${\mathbb {R}}^2$ into quantum states on ${\mathbb {C}}^2$ by means of (21). In this case, we observe that the performances of the Helstrom classifier are comparable to those of the linear SVM as expected^7^, except for the datasets *moons* and *prnn_synth* where the SVM turns out to be definitely more accuarate. However, for the *linearly_separable* dataset, Helstrom reaches a high average accuracy and for the datasets *analcatdata_boxing2* and *lupus* it is the most accurate classifier, with a tiny margin, over the classical and the quantum-inspired ones. In particular, for *analcatdata_boxing2*, Helstrom presents an average accuracy that is only 0.1% higher than the Bures’. The considered quantum-inspired nearest mean classifiers present comparable accuracies between them and w.r.t. Helstrom, except for *moons* and *prnn_synth* datasets where they definitely outperform Helstrom and for *circles* and *analcatdata_asbestos* datasets where the nearest mean classifiers present an average accuracy that is over 10% higher than Helstrom’s. Over the considered datasets, the nearest mean classifier based on the Bures distance turns out to be the quantum-inspired algorithm with the highest average accuracy for five datasets: *moons, linearly_separable, analcatdata_asbestos, Hill_Valley_with_noise, Hill_Valley_without_noise*.

Within the encoding of real data points into density matrices on ${\mathbb {C}}^3$, the performance of the Helstrom classifier gets better and approaches the average accuracy of the linear SVM over the *moons* and *prnn_synth* datasets (Table 3) and outperforms the linear SVM over the *cicles* dataset. Thus, within this encoding, the performance of Helstrom classifier over the considered datasets is comparable to that of the quantum-inspired nearest mean classifiers. The Euclidean and the Bures classifiers improve their accuracy for the *cicles* dataset. The considered three quantum-inspired classifiers worsen the already poor performance over the *analcatdata_aids* w.r.t. the lower-dimensional encoding. As shown in Table 4, increasing the dimension of the feature space, from 5 to 20, by the preparation of two copies of the quantum states in ${\mathbb {C}}^3\otimes {\mathbb {C}}^3$, the Helstrom classifier outperforms the linear SVM over *moons, cicles, linearly separable, lupus, prnn_synth* datasets presenting a comparable average accuracy to the SVM’s over the other datasets except for *analcatdata_aids* where the performances of the quantum-inspired classifiers remain poor. Considering higher dimensional feature spaces (Tables 5 and 6) the performances of the quantum-inspired classifiers improve except for the *analcatdata_aids* dataset where there is a worsening of average accuracy increasing the dimension. In particular, the Helstrom classifier improves its performance w.r.t. the linear SVM becoming a definitely more accurate classifier.

In Table 9, we observe that all the classifiers presents low values of average accuracy over the data set iii). However the *geometric Helstrom*, that is the classifier based on the minimum-error measurement determined by (13), performs better than the classical competitors, except Random Forest and Naive Bayes, and the classifier based on state discrimination by means of the Pretty Good measurement defined in (9). In particular, geometric Helstrom outperforms the Nearest neighbor classifier.

## Conclusions

The present paper is focused on some methods of quantum-inspired machine learning, in particular classification algorithms based on quantum state discrimination. We adopted a geometric approach in defining quantum encodings of classical data in terms of Bloch vectors of density operators. The geometry of quantum encoding has been analyzed in relation to the construction of feature maps and to the execution of the quantum-inspired classifiers. We considered algorithms based on the construction of an optimal measurement for state discrimination: the *Helstrom classifier* based on the well-known Helstrom’s theory of quantum discrimination^2^, a classifier based on the so-called *Pretty Good measurement*^10^ and a classifier based on the geometric construction of the minimum-error measurement^11^. Moreover we considered quantum-inspired nearest mean classifiers based on the encoding of data into density operators and the calculation of distances which quantify the distinguishability of quantum states in the spirit of other works on this subject^1,7^. The considered operator distances were: trace distance, Bures distance, Hellinger distance, Jensen–Shannon distance. The first two are particularly convenient in terms of the execution of a classifier within the *Bloch encoding* because the trace distance can be computed as the Euclidean distance among the Bloch vectors and the Bures distance allows the definition of a simple algorithm, reported in Algorithm 1, that perform the classification task entirely within the Bloch representation of the quantum states taking a full advantage by the geometric description. On the other hand, we do not find a satisfactory formulations of classification algorithms based on Hellinger and Jensen–Shannon distances that can executed entirely within the geometric description of the quantum states. Nevertheless, the experiments performed in the low-dimensional case (data encoding into qubit states) show that the classification done with the Hellinger and the Jensen–Shannon distances do not provide an average accuracy that is significantly different from that of the classifiers with trace and Bures distances, so we focused only on the latter for the experiments in higher dimension.

In “Geometric approach to quantum-inspired classifications” section, we clarified the adopted geometric approach. Within the encoding of real feature vectors into the amplitudes of pure quantum states w.r.t. a computational basis, the density operators are expressed as Bloch vectors and the centroids of data classes are directly calculated in terms of Bloch vectors. However, the mean of a set of Bloch vectors is not a Bloch vector in general (except in the case of qubit states). In order to identify the centroid as a proper density operator on ${\mathbb {C}}^d$ the obtained Bloch vector is re-scaled into the real sphere with radius $\frac{2}{d+1}$. The advantage in considering such a Bloch representation is given by data compression allowed suppressing null and repeated components in Bloch vectors removing redundancy in the representation. This simple property is useful when many copies of the considered quantum state $\rho$ are processed in order to increase the dimension of the feature space (kernel trick). In fact, the saving of spatial resources in representing $\rho \otimes \cdots \otimes \rho$ by means of the Bloch vectors balances the exponential cost due to processing the tensor product. Thus the Bloch representation turns out to be a useful tool to efficiently increase the dimension of the feature space in quantum-inspired machine learning.

In the experiments over different datasets, described in “Method and experimental results” section, the effects of the kernel tricks on the accuracy of the Helstrom classifier are evident. Moreover, the obtained results show that the performances of the quantum-inspired classifiers are comparable, and sometimes better, to those of well-known classical algorithms. We observed that the classification based on the minimum-error measurement for state discrimination can be carried on by the *Pretty Good measurement* or by the so-called *geometric Helstrom*. A comparison over the dataset *case-control study of esophageal cancer* show that the geometric Helstrom is definitely more accurate w.r.t. the classifier based on Pretty Good measurement. Moreover, in Table 9 the results show that geometric Helstrom outperforms also the classical support vectors machines, the KNN, and the logistic regression.

Description and characterization of the quantum-inspired classifiers considered in the present work suggest that quantum structures can be a valuable resource in classical machine learning, in particular the geometric approach considering the Bloch representation of density matrices is suitable to efficiently implement feature maps in quantum-inspired classification. The adopted geometric approach and the obtained experimental results reveal that quantum encoding of data into density operators and quantum state discrimination allow the definition of new efficient classification algorithms that can be run on classical computers.

## Data Availability

The code is also at the following repository: https://github.com/leporini/classification (accessed on: December 9, 2021).

## References

[1] Sergioli G, Bosyk G, Santucci E, Giuntini R (2017). A quantum-inspired version of the classification problem. Int. J. Theor. Phys..

[2] Helstrom C (1969). Quantum detection and estimation theory. J. Stat. Phys..

[3] Sergioli G, Giuntini R, Freytes H (2019). A new quantum approach to binary classification. PLoS One.

[4] Bertlmann, R. A. & Krammer, P. *Bloch Vectors for Qudits*. arXiv:0806.1174v1 (2008).

[5] Kimura G, Kossakowski A (2005). The Bloch-vector space for n-level systems: The spherical-coordinate point of view. Open Syst. Inf. Dyn..

[6] Giuntini, R. *et al.**Quantum State Discrimination for Supervised Classification*. arXiv:2104.00971v1 (2021).

[7] Leporini R, Pastorello D (2021). Support vector machines with quantum state discrimination. Quantum Rep..

[8] Croke S, Barnett S, Graeme W (2017). Optimal sequential measurements for bipartite state discrimination. Phys. Rev. A.

[9] Croke S, Andersson E, Barnett S, Gilson C, Jeffers J (2006). Maximum confidence quantum measurements. Phys. Rev. Lett..

[10] Mochon C (2006). Family of generalized pretty good measurements and the minimal-error pure-state discrimination problems for which they are optimal. Phys. Rev. A.

[11] Bae J (2013). Structure of minimum-error quantum state discrimination. New J. Phys..

[12] Freund Y, Schapire RE (1997). A decision-theoretic generalization of on-line learning and an application to boosting. J. Comput. Syst. Sci..

[13] Romano, J. *et al.**Pmlb v1.0: An Open Source Dataset Collection for Benchmarking Machine Learning Methods*. arXiv:2012.00058 (2020). 10.1093/bioinformatics/btab727 PMC8756190 34677586

